# Association of biomarkers of enteric dysfunction, systemic inflammation, and growth hormone resistance with seroconversion to oral rotavirus vaccine: A lasso for inference approach

**DOI:** 10.1371/journal.pone.0293101

**Published:** 2023-11-17

**Authors:** Katayi Mwila-Kazimbaya, Samuel Bosomprah, Obvious Nchimunya Chilyabanyama, Caroline Cleopatra Chisenga, Mwelwa Chibuye, Natasha Makabilo Laban, Michelo Simuyandi, Bert Huffer, Miren Iturriza-Gomara, Robert K. M. Choy, Roma Chilengi

**Affiliations:** 1 Research Division, Centre for Infectious Disease Research in Zambia, Lusaka, Zambia; 2 Department of Biostatistics, School of Public Health, University of Ghana, Accra, Ghana; 3 Department of Global Health, Amsterdam Institute for Global Health and Development (AIGHD), Amsterdam University Medical Centers, University of Amsterdam, Amsterdam, The Netherlands; 4 Cincinnati Childrens Hospital Medical Center, Cincinnati, Ohio, United States of America; 5 Centre for Vaccine Innovation and Access, PATH, Geneve, Switzerland; 6 PATH, Seattle, Washington, United States of America; Aga Khan University, PAKISTAN

## Abstract

**Background:**

Rotavirus gastroenteritis remains a leading cause of morbidity and mortality despite the introduction of vaccines. Research shows there are several factors contributing to the reduced efficacy of rotavirus vaccines in low- and middle-income settings. Proposed factors include environmental enteric dysfunction (EED), malnutrition, and immune dysfunction. This study aimed to assess the effect of these factors on vaccine responses using a machine learning lasso approach.

**Methods:**

Serum samples from two rotavirus clinical trials (CVIA 066 n = 99 and CVIA 061 n = 124) were assessed for 11 analytes using the novel Micronutrient and EED Assessment Tool (MEEDAT) multiplex ELISA. Immune responses to oral rotavirus vaccines (Rotarix, Rotavac, and Rotavac 5D) as well as a parenteral rotavirus vaccine (trivalent P2-VP8) were also measured and machine learning using the lasso approach was then applied to investigate any associations between immune responses and environmental enteric dysfunction, systemic inflammation, and growth hormone resistance biomarkers.

**Results:**

Both oral and parenteral rotavirus vaccine responses were negatively associated with retinol binding protein 4 (RBP4), albeit only weakly for oral vaccines. The parenteral vaccine responses were positively associated with thyroglobulin (Tg) and histidine-rich protein 2 (HRP2) for all three serotypes (P8, P6 and P4), whilst intestinal fatty acid binding protein (I-FABP) was negatively associated with P6 and P4, but not P8, and soluble transferrin receptor (sTfR) was positively associated with P6 only.

**Conclusion:**

MEEDAT successfully measured biomarkers of growth, systemic inflammation, and EED in infants undergoing vaccination, with RBP4 being the only analyte associated with both oral and parenteral rotavirus vaccine responses. Tg and HRP2 were associated with responses to all three serotypes in the parenteral vaccine, while I-FABP and sTfR results indicated possible strain specific immune responses to parenteral immunization.

## Introduction

Rotavirus remains a public health concern due to the high morbidity and mortality associated with rotavirus diarrhea [1]. Rotavirus vaccines have been successfully implemented globally, albeit with reduced efficacy in developing countries [2]. This was shown in a review conducted by Kazimbaya and colleagues, where the rotavirus vaccine demonstrated lower efficacy in low- and middle-income countries (LMICs) as compared to developed countries [3]. The review analyzed multiple efficacy trials for RotaTeq® and Rotarix®, conducted in high-income countries (HICs) such as the United States, Australia, Spain, Japan, Finland, and Canada and in these countries, the efficacy against hospitalization or emergency in-patient care of these vaccines was greater than 80% [4–9]. However, for reasons not well understood, these vaccines do not perform optimally in resource-poor country populations (LMICs), where the efficacy is between 40–60% [10–17]. Two other vaccines, ROTAVAC® and Rotasil®, have shown modest efficacy in their Phase 3 studies in India and Zambia [18–20]. The efficacy of ROTAVAC® against severe rotavirus diarrhea was 56.4% (95% CI 36.6–70.1) in India, while the efficacy estimates of Rotasil® was 39.5% (95% CI 26.7–50). The Zambian trial reported seroconversion rates of 33.1% whilst India reported seroconversion rates of 40.6% for ROTAVAC®. The similarity in seroconversion rated between Zambia and Indiacould indicate comparable efficacy rates for ROTAVAC® in Zambia [18, 20, 21].

Many reasons have been proposed to explain the observed differences in efficacy between LMICs and HICs, and these include interference by other viruses or bacteria colonizing the intestine, which impede the establishment of a protective immune response; neutralization of the vaccine virus by maternally derived antibody (from breast-milk or trans-placentally acquired antibody), immaturity of the local immune system; environmental enteric dysfunction (EED); interference from oral poliovirus vaccines; malnutrition and potential interference due to the concomitant presence of other pathogens [22–29].

Previous studies have observed both positive and non-significant relationships between the immune response to orally administered vaccinations with EED [22, 30, 31]. However, only one study tested the independent association of biomarkers of EED with other factors such as systemic inflammation and growth hormone resistance with seroconversion to oral rotavirus vaccination [30]. We used the 11-plex Micronutrient and EED Assessment Tool (MEEDAT) to measure biomarkers of EED, inflammation and growth resistance to ascertain the multiple factors that could be associated with vaccine seroconversion [30]. MEEDAT is intended to offer a rapid and effective tool for efficiently screening children for EED, systemic inflammation and growth failure prior to enrollment into clinical trials of candidate EED interventions. MEEDAT aims to streamline the evaluation of efficacy in trials for which the biomarkers are clinical endpoints. The performance of MEEDAT has been validated with well-qualified clinical specimens collected from children and adults in low-resource settings, a population at high risk for EED and stunting [30, 32, 33].

In this study, we aimed to investigate the association of biomarkers of EED, systemic inflammation, and growth hormone resistance at baseline with seroconversion to oral and parenteral rotavirus vaccination and linear growth using the machine learning (ML) lasso approach [34]. Parenteral vaccines have been predicted to have stronger immune responses and could show significant differences in immune profiles compared to oral vaccines and thus samples from both oral and parenteral vaccines were used in this study.

## Materials and methods

### Study design and participants

**Fig 1:**
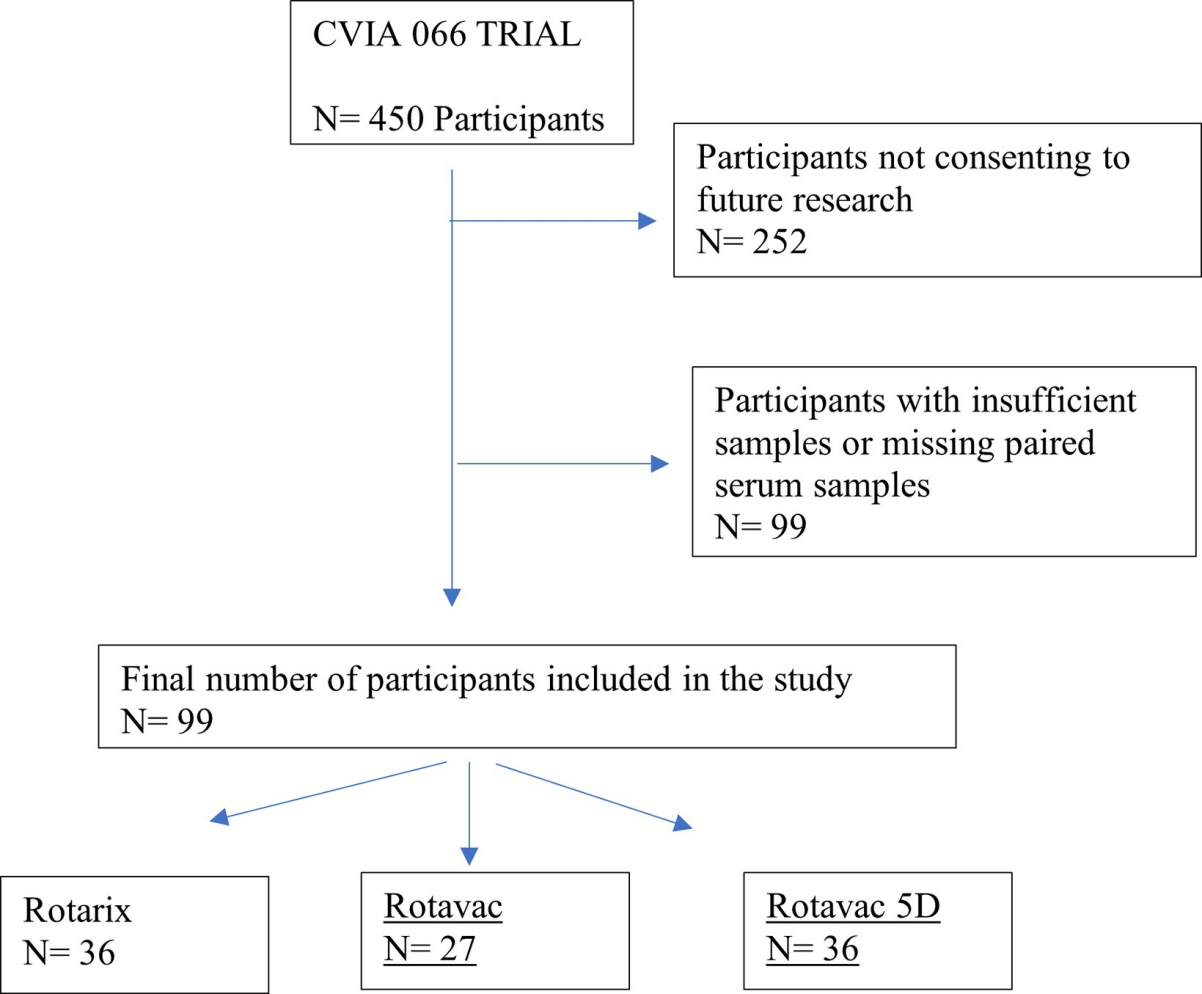
Sample selection from CVIA 066 trial.

Plasma samples were obtained on 14 December 2021 from 99 infants from the CVIA 066 trial (NCT03602053) and 124 infants from the CVIA 061 (NCT04010448). The CVIA 066 trial design and results were reported by Chilengi and colleagues [20] and the samples selected are shown in Fig 1. All samples obtained were deidentified with only participant IDs and no participant details were available to authors at any point during or after data collection.

**Fig 2:**
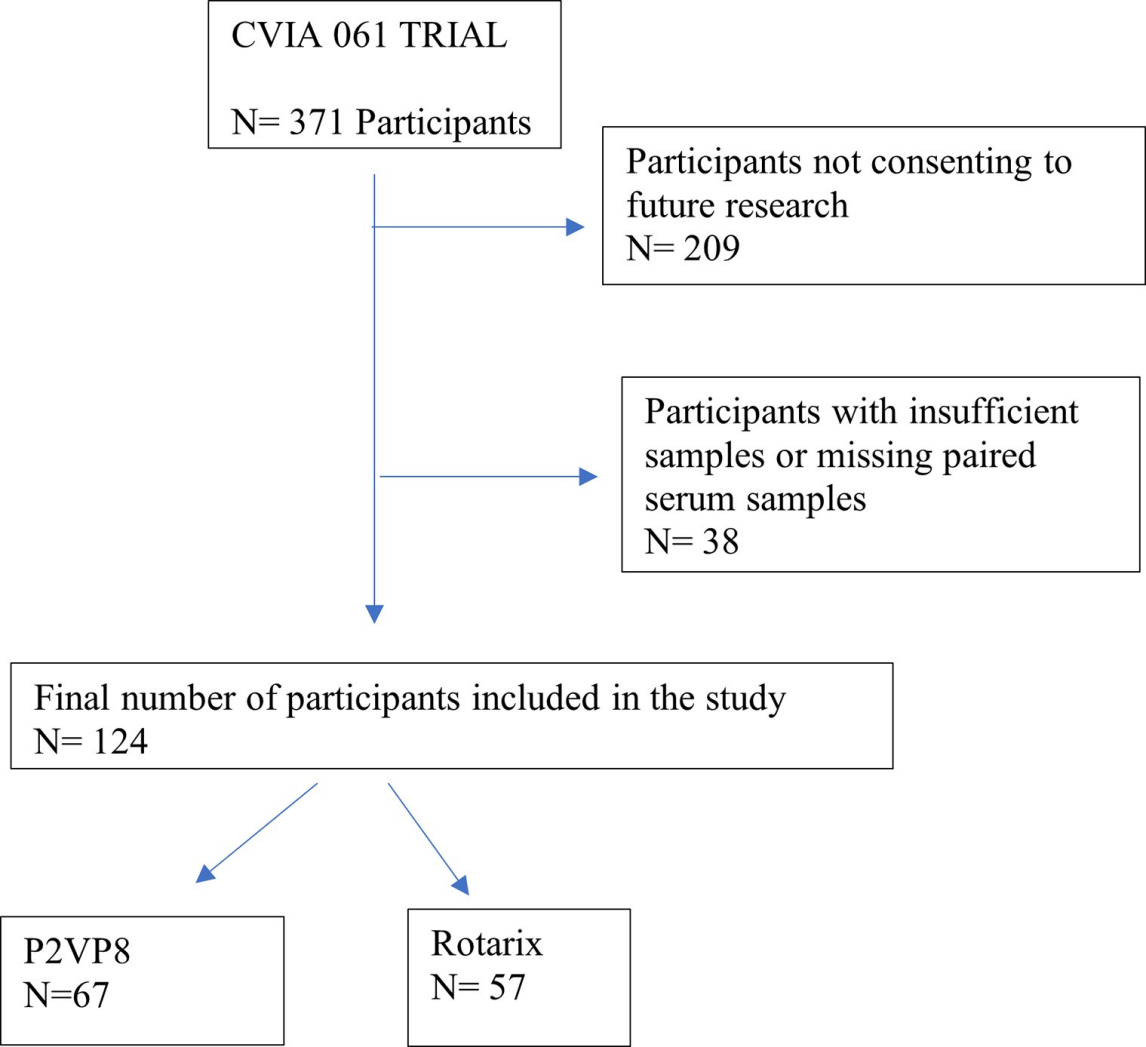
Sample selection from CVIA 061 trial.

The CVIA 061 trial is an ongoing Phase 3 double-blind, randomized, active comparator-controlled, group-sequential, multinational trial to assess the safety, immunogenicity, and efficacy of a trivalent (TV) rotavirus P2-VP8 subunit vaccine. A cohort of 371 healthy infants aged 6–8 weeks was randomized to one of two groups in a ratio of 1:1 to receive either 90 μg of TV rotavirus P2-VP8 subunit vaccine by intramuscular (IM) injection plus oral placebo, or Rotarix® PO plus IM placebo. Participants received three doses of TV P2-VP8/placebo IM and two doses of Rotarix®/placebo PO at monthly intervals starting at 6–8 weeks of age. To maintain the blind, infants allocated to the TV P2-VP8 vaccine arm received both TV P2-VP8 IM as well as oral placebo vaccine, and infants allocated to receive Rotarix® received both Rotarix® PO and placebo IM. The samples selected for this study are shown in Fig 2. All samples selected were from participants that consented to future testing of samples in CVIA 066 and CVIA 061.

### Study procedures

#### Specimen collection and preparation

Two serum samples were collected from infants, the first sample was collected at baseline just prior to vaccination and the second sample was collected 28 days post final vaccination. Blood samples of between 3–5 ml were collected from infants in serum separator tubes (SST) and were allowed to sit for 30 minutes prior to centrifugation at 1000g, aliquoted and transported to CIDRZ Central lab at 2–8°C for storage at -80°C until testing.

#### Quantitative measurement of EED biomarkers and biomarker testing

Duplicate samples were tested to determine serum levels of intestinal fatty acid binding protein (I-FABP), soluble CD14 (sCD14), insulin-like growth factor 1 (IGF-1), fibroblast growth factor 21 (FGF21), alpha-1 acid glycoprotein (AGP), C-reactive protein (CRP), ferritin, soluble transferrin receptor (sTfR), retinol binding protein 4 (RBP4), thyroglobulin (Tg) and histidine-rich protein 2 (HRP2) as measured by the MEEDAT 11-plex ELISA (Q-Plex™ Human Environmental Enteric Dysfunction 11-plex, Quansys Biosciences, USA) as described by Arndt and colleagues [30]. All samples and calibrator dilutions were diluted using the reconstituted competitor as per the manufacturer’s instructions. All curve fitting was automatically completed using Quansys’ Q-View™ software, application version 3.11. Each analyte within the assay was validated with the MEEDAT array to meet the intra- and inter-assay specifications of <10% and <15% coefficient of variation (CV) respectively.

#### Measurement of immune response to vaccine antigens

All serum samples collected under the CVIA 066 study were tested for anti-rotavirus immunoglobulin A (IgA) antibodies by a validated ELISA using WC3 virus as the antigen at the Wellcome Trust Research Laboratory, Christian Medical College (CMC), Vellore, India. The assays had acceptable accuracy, precision, and linearity, with a standard curve modelled using a 4-parameter logistic fit regression function. During validation of the assay, the lower limit of quantification was set at 7.0 U/mL at CMC [20]. The ELISAs used to test the CVIA 061 study TV P2-VP8 samples used the WC3 lysate to assess anti P2_VP8 IgG antibodies and thus serum samples were shipped to Cincinnati Children’s Hospital Medical Center (CCHMC), Cincinnati, USA for analysis [35]. Both laboratories used assays developed in conformance with the WHO *Manual of Rotavirus Detection and Characterization Methods* [36]

### Data management

Results from the MEEDAT analysis were exported using Quansys’ Q-View software, application version 3.11. All results were optimized for best fit curves for individual biomarkers controls prior to exportation as CSV files.

### Ethics

A waiver of consent was obtained for the samples used in this study from the University of Zambia Bioethics Research Committee (UNZABREC ref 1977–2021) as all participants included had consented to future testing of samples. Additionally, waivers were obtained from Zambia Medicines Regulatory Authority (ZAMRA) and National Health Research Authority (NHRA). All study procedures were conducted in accordance with Good Clinical Practice.

### Statistical analysis

The primary outcome was vaccine response and was measured as seroconversion in the entire CVIA 066 cohort and CVIA 061 infants that received Rotarix. Seroconversion was defined as a four-fold increase in serum anti-rotavirus IgA antibody concentrations at day 28 post final dose. For the CVIA 061 cohort, IgG concentration levels for antigens P8, P6 and P4 titres in TV P2-VP8 samples were measured at day 28 post final dose.

We summarized baseline characteristics of participants using frequency and proportion for categorical variables, and median and interquartile range for continuous variables. To keep a variable with high variability from dominating the analysis, we standardized the biomarkers, P8, P6, P4 and serum anti-rotavirus IgA antibody concentrations to a mean of 0, a standard deviation of 1. We used heat plots to visualise the uniformity of biomarkers of enteric dysfunction, systemic inflammation, and growth hormone resistance at baseline and serum anti-rotavirus IgA antibody concentrations at day 28 post final dose. We used a cross-fit partialling-out logit model to estimate the effect of biomarkers on seroconversion controlling for social demographic characteristics and to determine the effect of biomarkers on P8, P6 and P4. Cross-fit partialling-out estimation methods overcome overfitting by orthogonalizing the relevant estimators [37]. The lasso for inference was chosen over the classical logistic regression because of the high dimensionality of the data. Therefore, constraining/regularising the objective or optimisation function in the lasso for inference helped to ensure stability of the estimates. All analyses were carried out using Stata 18 (StataCorp, College Station, TX, USA).

## Results

### Baseline characteristics of infants

A total of 99 infants were included for the CVIA 066 study and 57 infants in the oral arm (Rotarix) of the CVIA 061 study. Sixty-seven (~43%) were females and 21(13.5%) stunted defined as HAZ >-2 (Table 1).

**Table 1:** Baseline characteristics of infants in the CVIA 066 and CVIA 061 studies who received oral rotavirus vaccine.

	N(% of total)	Rotarix	Rotavac	Rotavac 5D	Chi2 p value
Sex					
Female	67(42.9)	38(57)	17(25)	12(18)	
Male	89(57.1)	55(62)	10(11)	24(27)	0.063
Age group (Weeks)					
<7	138(88.5)	84(61)	23(17)	31(22)	
7+	18(11.5)	9(50)	4(22)	5(28)	0.933
Stunted					
No	135(86.5)	84(62)	21(16)	30(22)	0.547
Seroconversion					
Yes	100(64.1)	56(56)	20(20)	24(24)	0.228

Table 2 indicates the baseline characteristics for 67 participants that were included in the CVIA 061 TV P2-VP8 arm of the study. Approximately 51% were male and 6% were stunted in the cohort.

**Table 2:** Baseline characteristics for participants enrolled in CVIA 061 study that received parenteral rotavirus vaccine.

		P8 Titres		P6 Titres		P4 Titres	
	N(% of total), N = 67	median (IQR)	*p-value	median (IQR)	*p-value	median (IQR)	*p-value
Age							
<7 weeks	61 (91%)	92.3 (45.6, 256.1)		181.1 (62.4, 689.2)		4522.6 (2604.1, 6895.9)	
7+ weeks	6 (9%)	106.25 (32.7, 159.5)	0.52	119.85 (23.5, 183.6)	0.47	3516.2 (2636.75, 10492.4)	0.87
Sex							
Male	34 (51%)	120.2 (40.8, 236.2)	0.65	206.2 (59.4, 596.2)	0.9	4097.4 (2146.5, 7585)	0.89
Female	33 (49%)	62.8 (46.8, 256.1)		113.4 (62.4, 943.7)		4684.75 (2674.65, 5994.2)	
Stunting							
No	63 (94%)	92.3 (41.5, 256.1)	0.56	177.7 (52.4, 630)	0.33	4097.4 (2604.1, 6895.9)	0.53
Yes	4 (6%)	84.4 (41.4, 135.8)		560.65 (126.55, 1054.45)		5682.4 (4522.6, 6842.2)	

* Wilcoxon rank sum test

We observed the day 28 responses differed by participants. Likewise, biomarker concentration strength differs by participants and biomarker, and notably some participants exhibited relatively higher levels across multiple biomarkers. We also observed the relationships between the baseline biomarker levels and immune response by subject, e.g., a group of subjects in the oral rotavirus arm with strong responses appear to have high baseline levels of biomarkers compared to those in the parenteral arm (S1 and S2 Figs compared to S3 Fig). To better extract, assess, and utilize such observations, machine learning techniques were applied to provide models of the relationship between biomarkers of environmental enteric dysfunction, systemic inflammation, and growth hormone resistance at baseline and rotavirus-specific antibodies induced by vaccination (Table 3).

**Table 3:** Association of biomarkers of environmental enteric dysfunction, systemic inflammation, and growth hormone resistance at baseline with seroconversion to oral rotavirus vaccination (Rotarix, Rotavac and Rotavac 5D) among infants aged 6–10 weeks at vaccination for CVIA 066 and CVIA 061 participants.

Biomarkers	*Adjusted OR	95%CI		p-value
Ferritin	0.94	[0.62,	1.44]	0.79
Tg	1.06	[0.71,	1.6]	0.76
sTfR	0.79	[0.51,	1.21]	0.28
HRP2	1.12	[0.75,	1.67]	0.59
IGF-1	1.2	[0.86,	1.68]	0.28
FGF21	0.88	[0.61,	1.27]	0.49
I-FABP	1.28	[0.66,	2.48]	0.46
CRP	1.37	[0.9,	2.08]	0.14
sCD14	1.31	[0.68,	2.53]	0.42
AGP	0.99	[0.57,	1.71]	0.96
RBP4	0.53	[0.26,	1.08]	0.08

*Cross-fit partialing-out logit model adjusted for age in weeks (binary), sex (binary), low birth weight (binary), stunted (binary). Biomarker concentrations were standardised to mean of 0 and standard deviation of 1. The adjusted odds ratio (aOR) corresponds with a 1 standard deviation increase in biomarker.

### Association of biomarkers at baseline with seroconversion to oral rotavirus vaccine

Cross-fit partialing-out logit model, a lasso for inference machine learning algorithm, showed that only RBP4 (p = 0.08) was weakly associated with seroconversion (Table 3).

### Association of biomarkers at baseline with vaccine response to parenteral rotavirus vaccine

Cross-fit partialing-out linear model for the CVIA 061 participants that received TV P2-VP8, a lasso for inference machine learning algorithm, showed that HRP2 (p = 0.06) was weakly associated with increased P8 antigen titres post vaccination and Tg (p = 0.008) had a stronger association for P8 antigen titres post vaccination, whilst RBP4 (p = 0.013) had a stronger negative association of post vaccination titre with the P8 antigen (Table 4) whilst only Tg (0.041) and RBP4 (-0.048) showed an association for change of titre with the P8 antigen (Table 5).

**Table 4:** Association of biomarkers of environmental enteric dysfunction, systemic inflammation, and growth hormone resistance to post vaccination titres of P8.

P8 Antigen Titre Post vaccination				
Biomarkers	*Adjusted Coefficient	95%CI		p-value
Ferritin	-0.07	[-0.3,	0.16]	0.541
Tg	0.25	[0.06,	0.43]	**0.008**
sTfR	0.18	[-0.13,	0.49]	0.255
HRP2	0.33	[-0.01,	0.67]	**0.06**
IGF-1	0	[-0.22,	0.22]	0.993
FGF21	-0.02	[-0.2,	0.16]	0.828
I-FABP	-0.1	[-0.3,	0.09]	0.286
CRP	0.37	[-0.05,	0.8]	0.086
sCD14	0.39	[-0.05,	0.84]	0.081
AGP	-0.22	[-0.61,	0.18]	0.283
RBP4	-0.57	[-1.01,	-0.12]	**0.013**

*Cross-fit partialing-out logit model adjusted for age in weeks (binary), sex (binary), low birth weight (binary), stunted (binary). Biomarker concentrations were standardised to mean of 0 and standard deviation of 1

**Table 5:** Association of biomarkers of environmental enteric dysfunction, systemic inflammation, and growth hormone resistance to change in antigen titre of P8.

P8 Change in Antigen Titre				
Biomarkers	*Adjusted Coefficient	95%CI		p-value
Ferritin	-0.11	[-0.36,	0.13]	0.377
Tg	0.2	[0.01,	0.39]	**0.041**
sTfR	0.25	[-0.02,	0.53]	0.073
HRP2	0.29	[-0.07,	0.64]	0.11
IGF-1	0	[-0.22,	0.21]	0.975
FGF21	0.01	[-0.16,	0.17]	0.933
I-FABP	-0.17	[-0.37,	0.02]	0.084
CRP	0.36	[-0.03,	0.75]	0.074
sCD14	0.24	[-0.21,	0.68]	0.294
AGP	-0.19	[-0.57,	0.19]	0.323
RBP4	-0.48	[-0.96,	0]	**0.048**

For the P6 the biomarkers Tg (p = 0.036), sTfR (p = 0.003), HRP2 (p = 0.038) showed a positive association whilst I-FABP (p = 0.015) and RBP4 (p = 0.037) showed negative associations with post vaccination titre (Table 6) whilst sTfR (p<0.001) showed strong association with change in titre and HRP2 (p = 0.062), I-FABP (p = 0.02), Tg (p = 0.012) and RBP4 (p = 0.068) showed weaker associations with changing titre of P6 antigen (Table 7).

**Table 6:** Association of biomarkers of environmental enteric dysfunction, systemic inflammation, and growth hormone resistance to post vaccination titres of P6.

P6 Antigen Titre Post vaccination				
Biomarkers	*Adjusted Coefficient	95%CI		p-value
Ferritin	0.08	[-0.14,	0.29]	0.472
Tg	0.16	[0.01,	0.31]	**0.036**
sTfR	0.59	[0.2,	0.98]	**0.003**
HRP2	0.3	[0.02,	0.57]	**0.038**
IGF-1	0.13	[-0.06,	0.33]	0.183
FGF21	0.09	[-0.15,	0.33]	0.442
I-FABP	-0.32	[-0.58,	-0.06]	**0.015**
CRP	0.07	[-0.13,	0.27]	0.512
sCD14	0.23	[-0.32,	0.78]	0.417
AGP	-0.13	[-0.49,	0.22]	0.459
RBP4	-0.46	[-0.9,	-0.03]	**0.037**

**Table 7:** Association of biomarkers of environmental enteric dysfunction, systemic inflammation, and growth hormone resistance to change in antigen titre of P6.

P6 Change in Antigen Titre					
Biomarkers	*Adjusted Coefficient	95%CI			p-value
Ferritin	0.13	[-0.1,	0.35]		0.277
Tg	0.21	[0.05,		0.38]	**0.012**
sTfR	0.59	[0.26,		0.92]	**<0.001**
HRP2	0.24	[-0.01,		0.49]	**0.062**
IGF-1	0.16	[-0.04,		0.36]	0.116
FGF21	0.17	[-0.06,		0.39]	0.155
I-FABP	-0.3	[-0.56,		-0.05]	**0.02**
CRP	0.15	[-0.02,		0.32]	0.091
sCD14	0.08	[-0.41,		0.58]	0.745
AGP	-0.23	[-0.57,		0.11]	0.178
RBP4	-0.38	[-0.78,		0.03]	**0.068**

For P4 antigen the Tg (p = 0.005) HRP2 (p = 0.009) I-FABP (p = 0.061) and RBP4 (p = 0.007) showed an association for post vaccination titre (Table 8) whilst Tg (p = 0.026) HRP2 (p = 0.037) I-FABP (p = 0.014) and RBP4 (p = 0.047) also showed an association with the change in titre of P4 antigen (Table 9).

**Table 8:** Association of biomarkers of environmental enteric dysfunction, systemic inflammation, and growth hormone resistance to post vaccination titres of P4.

P4 Antigen Titre Post vaccination				
Biomarkers	*Adjusted Coefficient	95%CI		p-value
Ferritin	-0.06	[-0.3,	0.18]	0.621
Tg	0.3	[0.09,	0.5]	**0.005**
sTfR	0.16	[-0.13,	0.45]	0.29
HRP2	0.35	[0.09,	0.62]	**0.009**
IGF-1	0.08	[-0.17,	0.33]	0.525
FGF21	0.04	[-0.14,	0.22]	0.672
I-FABP	-0.19	[-0.39,	0.01]	**0.061**
CRP	0.27	[-0.02,	0.55]	0.069
sCD14	0.38	[-0.1,	0.86]	0.122
AGP	-0.19	[-0.61,	0.22]	0.362
RBP4	-0.54	[-0.93,	-0.15]	**0.007**

**Table 9:** Association of biomarkers of environmental enteric dysfunction, systemic inflammation, and growth hormone resistance to change in antigen titre.

P4 Change in Antigen Titre				
Biomarkers	*Adjusted Coefficient	95%CI		p-value
Ferritin	-0.1	[-0.35,	0.15]	0.42
Tg	0.25	[0.03,	0.46]	**0.026**
sTfR	0.21	[-0.06,	0.48]	0.127
HRP2	0.31	[0.02,	0.61]	**0.037**
IGF-1	0.06	[-0.17,	0.3]	0.594
FGF21	0.07	[-0.1,	0.23]	0.423
I-FABP	-0.26	[-0.47,	-0.05]	**0.014**
CRP	0.25	[-0.02,	0.52]	0.071
sCD14	0.24	[-0.23,	0.71]	0.308
AGP	-0.16	[-0.55,	0.23]	0.433
RBP4	-0.45	[-0.89,	-0.01]	**0.047**

To compare the parenteral and oral vaccine responses post vaccination, a similar analysis was carried out with oral vaccine response from CVIA 066 and CVIA 061 in which an adjusted coefficient was assessed instead of using seroconversion (odds ratio). The results in Table 10 indicated CRP and RBP4 were significant for oral vaccines (P<0.001 and P = 0.043).

**Table 10:** Association of biomarkers of environmental enteric dysfunction, systemic inflammation, and growth hormone resistance to post vaccination IgA titre of oral rotavirus vaccine.

Oral IgA Titre Post Vaccination				
	*Adjusted Coefficient	95 CI Interval		p-value
Ferritin	-0.097	[-0.366,	0.173]	0.482
Tg	-0.01	[-0.163,	0.143]	0.897
sTfR	-0.043	[-0.308,	0.221]	0.747
HRP2	-0.155	[-0.37,	0.059]	0.156
IGF-1	0.119	[-0.157,	0.395]	0.399
FGF21	0.088	[-0.154,	0.329]	0.476
I-FABP	0.029	[-0.239,	0.296]	0.832
CRP	0.295	[0.158,	0.431]	**<0.001**
sCD14	-0.1	[-0.405,	0.205]	0.521
AGP	0.208	[-0.173,	0.589]	0.285
RBP4	-0.21	[-0.413,	-0.006]	**0.043**

A comparison was also made of the analyte ranges amongst the participants of the Zambian trials compared to the Tanzanian [33] and Malian cohorts [30] that also used MEEDAT to assess the nutritional, growth and EED status of infants (Table 11). Most of the analytes were similar amongst the infants with the Zambian infants generally having lower biomarker concentrations. The exceptions noted were ferritin, IGF-1, sTfR and Tg for the Malian infants and only FGF21 for the Tanzanian infants.

**Table 11:** Comparison of analyte biomarker levels in Zambian infants (n = 156), Tanzanian infants (n = 365) and Malian infants (n = 299).

Serum Analyte	ZAMBIAN			TANZANIAN*			MALIAN^%^	
	Median	IQR	Median			IQR	Median	IQR
AGP (g/L)	0.71	[0.52,	1.045]		0.83	[0.61, 1.26]	0.85	[0.68, 1.09]
sCD14 (ng/ml)	1498.55	[1044.105,	2207.32]		1829.46	[1404.7, 2287.57]	1797.7	[1429.7, 2159.9]
CRP (mg/L)	0.29	[0.11,	0.685]		0.36	[0.00, 4.40]	0.65	[0.30, 2.65]
Ferritin (μg/L)	501.10	[256.23,	858.145]				8.89	[3.30, 24.43]
FGF21(pg/ml)	167.28	[96.5,	422.62]		88.90	[47.94, 174.50]	164.6	[96.2, 345.7]
HRP2 (μg/L)	0.01	[0.007,	0.013]				0.006	[0.006, 0.009]
I-FABP (pg/ml)	903.47	[541.59,	1323.04]		1094.62.1	[732.21, 1634.80]	1052.1	[697.4, 1493.2]
IGF-1(ng/ml)	40.13	[12.835,	62.765]		66.48	[32.24, 117.92]	18.2	[9.7, 30.6]
RBP4 (μmol/L)	2.06	[1.48,	4.25]				1.56	[1.30, 1.87]
sTfR (mg/L)	9.20	[7.31,	11.965]				20.26	[15.18, 31.12]
Tg (μg/L)	100.94	[69.415,	139.15]				45.85	[29.85, 75.38]

* [33]

^%^ Comparison data from Malian cohort already published [30]

## Discussion

We report associations between two rotavirus vaccines and biomarkers of growth hormone resistance, environmental enteric dysfunction and systemic inflammation. For the oral vaccine recipients (Rotarix, Rotavac and Rotavac 5D), RBP4 (p = 0.08) showed a weak negative association with seroconversion and in comparison, RBP4 (p = 0.043) and CRP (p<0.001) showed a negative and positive association respectively with parenteral vaccine immune response. CRP has been associated with immune activation and acute or chronic inflammation and RBP4 is also associated with immune activation and micronutrient deficiency. RBP4 has been proposed as an indirect measure of nutrition and vitamin A deficiency and vitamin A has been found to contribute to disease severity in its absence. Thus, a downward change in RBP4 could indicate reduced immune activation, improved nutrition and thus possibly facilitate towards seroconversion. CRP showed a positive association with seroconversion and therefore increase in CRP could also assist in seroconversion of infants. It was noted that none of the environmental enteric dysfunction markers were associated with seroconversion, though this may be explained by the sample size being insufficiently powered to detect a significant change.

For the parenteral vaccine TV P2-VP8, RBP4, Tg and HRP2 were the biomarkers commonly associated with both post vaccination titres as well as change in titres for all antigens. Tg can indirectly measure the nutritional status of infants as higher levels indicate that the thyroid may be overcompensating for low levels of iodine and could indicate iodine deficiency. Normal function of thyroid is necessary for brain development and has also been found to impact T-cell function [38]. Therefore, increased levels could potentially impact the ability of an infant to successfully respond to a parenteral vaccine and may account for its marked association with vaccination titres post vaccination and changes in titres post vaccination.

For the parenteral vaccines there was a difference seen amongst the antigens with I-FABP and sTfR being associated with P6 antigens and I-FABP being associated with titres against the P4 antigens respectively in addition to RBP4, Tg and HRP2. Soluble transferrin receptor has been identified as another micronutrient status indicator as it measures iron deficiency. Low iron deficiency has been linked to poor growth outcomes for infants globally and would have a long-term impact on immune status. It was observed that for P6 and P4 antigens only one of the environmental enteric dysfunction markers (I-FABP) an indicator of gut repair which is associated with stunting and growth faltering had a strong negative association. It is not clear why this would not apply to all antigens but may point to different mechanisms of action. An analysis carried out by Cates and colleagues indicated that vaccine effectiveness differed by strain, with a larger difference observed in LMICs [39]. The phase II study carried out in South Africa by Groome and colleagues noted that infants showed immune responses between 77–85% for two strains and 52–65% responses to all three strains therefore showing that there were strain specific responses amongst the infants [40]. They also showed differences in IgA immune responses with the three antigens with P8 showing the smallest responses though no reason was proposed for this observation [40]. Our study also showed the least responses for the P8 antigens, therefore, it may be that environmental factors in LMICs may account for the differences observed amongst the antigens with P6 and P4 producing greater immune responses as compared to P8 in the parenteral vaccine.

Previous work in Zambian infants by Mwape and colleagues [22] identified zonulin and I-FABP as associated with increased response to rotavirus vaccine. Whilst work in Nicaragua associated myeloperoxidase and calprotectin levels with failed seroconversion and generally higher combined EED scores also with failure to seroconvert [41]. Additional work in Zimbabwe determined that I-FABP as well as myeloperoxidase were positively associated with rotavirus vaccine immune response [31]. To the contrary this study was able to identify a negative association between I-FABP and increased titres of P6 and P4 antigens in the TV P2-VP8 cohort, it was also able to identify other EED, systemic inflammation and growth biomarkers that may be associated with vaccine response. The study made use of the machine learning (ML) lasso approach, a more statistically advanced method compared to traditional methods.

The difference observed in the biomarkers may give us some indication of the differences in immune responses between oral vaccines and parenteral vaccines though RBP4 which was associated with both types of vaccines may provide further research possibilities to better understand differences. RBP4 has been identified as a proxy for vitamin A deficiency which is well known for its association with increased disease severity and reduced immune response and could potentially explain the negative correlation with immune responses observed against both oral and parenteral vaccines [42]. Tg, HRP2 and I-FABP were associated with parenteral immune responses. This points to the differences that the oral and immune responses were eliciting, and further investigations would assist in more clearly understanding the pathways for these responses. The causes for reduced efficacy are likely multifactorial, and their identification could allow the design of strategies for vaccine improvement. Due to the high burden of rotavirus disease, even a modest improvement in vaccine effectiveness in the individual could nonetheless have a significant overall public health impact. This study was able to successfully identify biomarkers of environmental enteric dysfunction, growth faltering and inflammation that could potentially be impacting the immune responses of infants in oral and parenteral vaccination and could provide further areas of research as we better seek to find more effective vaccines for rotavirus. The study was also able to utilize MEEDAT to effectively measure and analyze biomarker levels in a different population to those previously assessed.

A major limitation of this study was the sample size used, more so for the parenteral TV P2-VP8 vaccine. To be able to generalize the results observed would require additional testing with larger sample sets. Additionally, the MEEDAT is a novel method of analysis that was employed and was able to show similarities in biomarker levels in Zambian, Malian and Tanzanian infants. The Zambian infant cohort was able to successfully show the increased capacity (range) of the MEEDAT in measurement of biomarkers. The comparison was also able to show that despite Zambia, Mali and Tanzania being sub-Saharan there were some differences in the cohorts as shown by significant differences in ferritin, IGF-1, sTfR, FGF21 and Tg levels between the three countries. The Zambian infants appear to be slightly healthier compared to their Malian counterparts as evidenced by the increased levels of ferritin, IGF-1, sTfR and Tg which indicate healthier immune status and micronutrition as well as improved growth hormones. This may however be explained by the fact that the Tanzanian cohort of children were all HIV exposed whereas the Zambian infants were mixed and the Malian infants were slightly older than the Zambian cohort. Another additional limitation is the study only tested the vaccination time points, testing of follow up diarrheal surveillance samples may have helped to elucidate further links between the biomarkers, vaccine responses and immune protection. Similarly data on concomitant infections may also have revealed further linkages.

## Supporting information

S1 Fig Input data for each of 99 CVIA 066 and 57 CVIA 061 participants receiving oral rotavirus vaccine (per row), measurements of biomarkers of enteric dysfunction, systemic inflammation, and growth hormone resistance at baseline. The heatmap colours indicate concentration within each column, standardized to a mean of 0, a standard deviation of 1, and truncated at 6σ.
(TIF)

S2 Fig Input data for each of 99 CVIA 066 and 57 CVIA 061 participants receiving oral rotavirus vaccine per vaccine type (per row), measurements of biomarkers of enteric dysfunction, systemic inflammation, and growth hormone resistance at baseline. The heatmap colours indicate concentration within each column, standardized to a mean of 0, a standard deviation of 1, and truncated at 6 σ.
(TIF)

S3 Fig Input data for each of 67 CVIA 061 participants receiving parenteral rotavirus vaccine (per row), measurements of biomarkers of enteric dysfunction, systemic inflammation, and growth hormone resistance at baseline. The heatmap colours indicate concentration within each column, standardized to a mean of 0, a standard deviation of 1, and truncated at 6σ.
(TIF)
