# Supplementary figures and images for: Association of biomarkers of enteric dysfunction, systemic inflammation, and growth hormone resistance with seroconversion to oral rotavirus vaccine: A lasso for inference approach
Source: PLoS One. 2023 Nov 17;18(11):e0293101. doi: 10.1371/journal.pone.0293101 (PMC10656027; doi:10.1371/journal.pone.0293101)

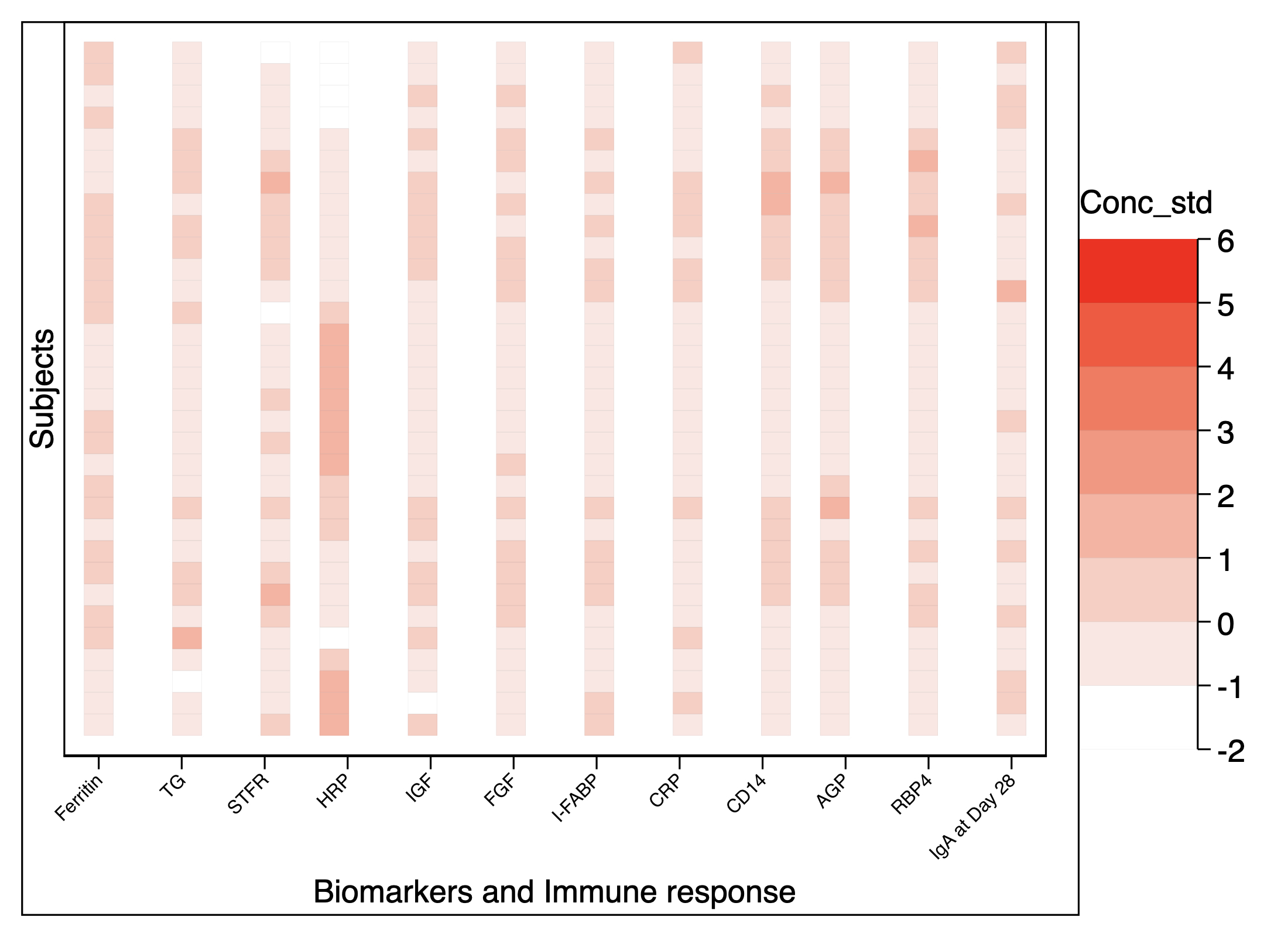

Supplement: S1 Fig — The heatmap colours indicate concentration within each column, standardized to a mean of 0, a standard deviation of 1, and truncated at 6σ. (TIF) [file pone.0293101.s001.tif]

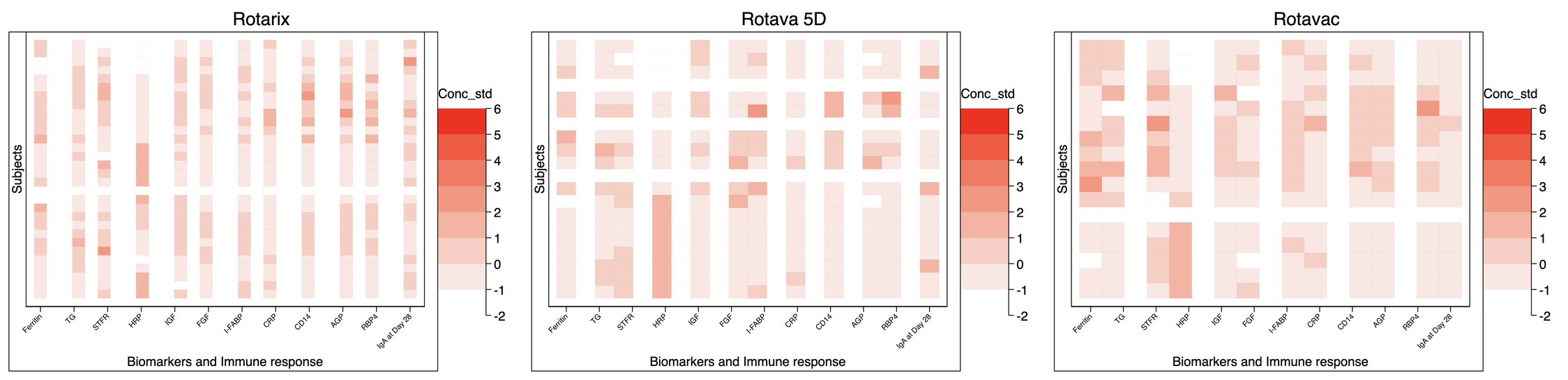

Supplement: S2 Fig — The heatmap colours indicate concentration within each column, standardized to a mean of 0, a standard deviation of 1, and truncated at 6 σ. (TIF) [file pone.0293101.s002.tif]

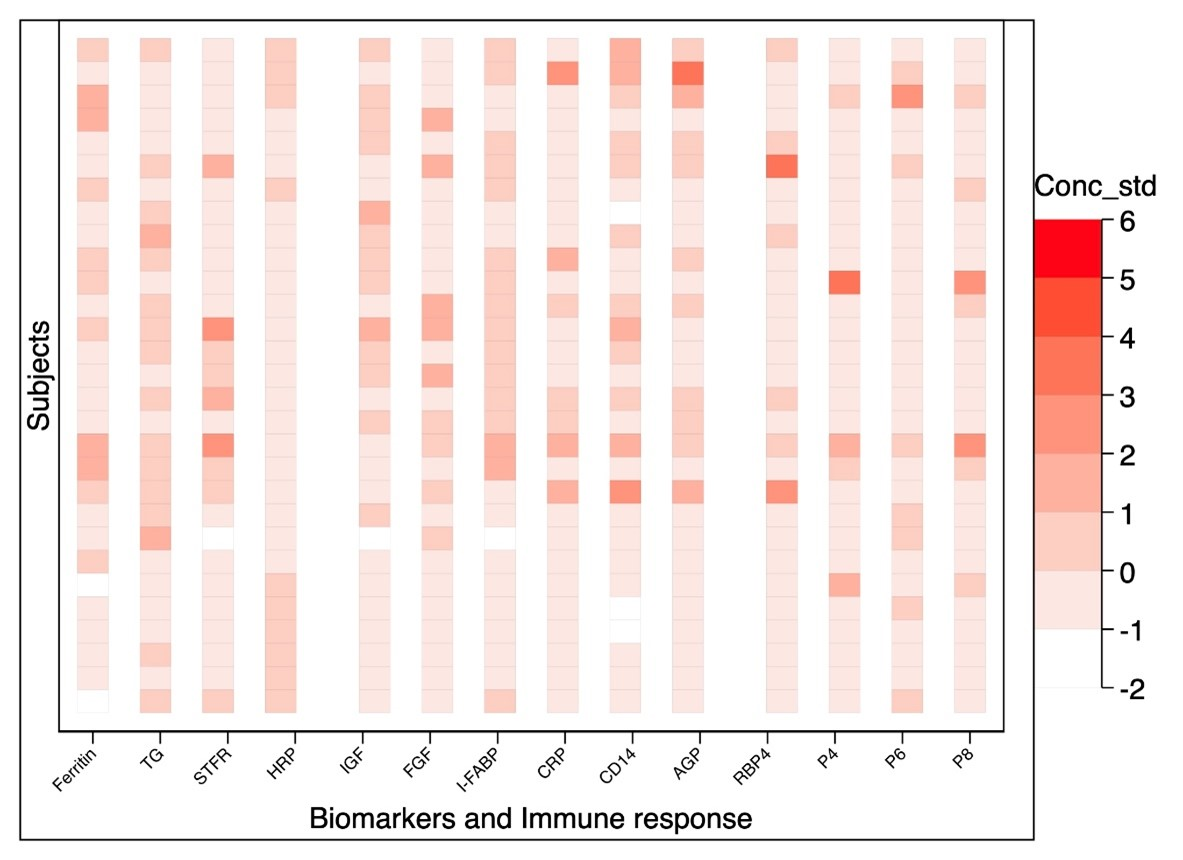

Supplement: S3 Fig — The heatmap colours indicate concentration within each column, standardized to a mean of 0, a standard deviation of 1, and truncated at 6σ. (TIF) [file pone.0293101.s003.tif]
